# Anisotropic Chitosan Scaffolds Generated by Electrostatic Flocking Combined with Alginate Hydrogel Support Chondrogenic Differentiation

**DOI:** 10.3390/ijms22179341

**Published:** 2021-08-28

**Authors:** Elke Gossla, Anne Bernhardt, Robert Tonndorf, Dilbar Aibibu, Chokri Cherif, Michael Gelinsky

**Affiliations:** 1 Centre for Translational Bone, Joint and Soft Tissue Research, University Hospital and Faculty of Medicine, Technische Universität Dresden, D-01307 Dresden, Germany; elke.gossla@gmail.com (E.G.); michael.gelinsky@tu-dresden.de (M.G.); 2 Institute of Textile Machinery and High Performance Material Technology, Technische Universität Dresden, D-01062 Dresden, Germany; robert.tonndorf@tu-dresden.de (R.T.); dilbar.aibibu@tu-dresden.de (D.A.); chokri.cherif@tu-dresden.de (C.C.)

**Keywords:** chitosan, electrostatic flocking, cartilage, alginate, chondrocytes

## Abstract

The replacement of damaged or degenerated articular cartilage tissue remains a challenge, as this non-vascularized tissue has a very limited self-healing capacity. Therefore, tissue engineering (TE) of cartilage is a promising treatment option. Although significant progress has been made in recent years, there is still a lack of scaffolds that ensure the formation of functional cartilage tissue while meeting the mechanical requirements for chondrogenic TE. In this article, we report the application of flock technology, a common process in the modern textile industry, to produce flock scaffolds made of chitosan (a biodegradable and biocompatible biopolymer) for chondrogenic TE. By combining an alginate hydrogel with a chitosan flock scaffold (CFS+ALG), a fiber-reinforced hydrogel with anisotropic properties was developed to support chondrogenic differentiation of embedded human chondrocytes. Pure alginate hydrogels (ALG) and pure chitosan flock scaffolds (CFS) were studied as controls. Morphology of primary human chondrocytes analyzed by cLSM and SEM showed a round, chondrogenic phenotype in CFS+ALG and ALG after 21 days of differentiation, whereas chondrocytes on CFS formed spheroids. The compressive strength of CFS+ALG was higher than the compressive strength of ALG and CFS alone. Chondrocytes embedded in CFS+ALG showed gene expression of chondrogenic markers (*COL II, COMP, ACAN*), the highest collagen II/I ratio, and production of the typical extracellular matrix such as sGAG and collagen II. The combination of alginate hydrogel with chitosan flock scaffolds resulted in a scaffold with anisotropic structure, good mechanical properties, elasticity, and porosity that supported chondrogenic differentiation of inserted human chondrocytes and expression of chondrogenic markers and typical extracellular matrix.

## 1. Introduction

The treatment of damaged or degenerated articular cartilage tissue has great scientific but also medical and economic relevance since (unlike other tissues) non-vascularized articular cartilage has a very limited self-healing capacity. Therefore, the therapy of cartilage damage using tissue engineering is a promising treatment option.

In common cartilage tissue engineering (TE) approaches, human chondrocytes are obtained from unloaded and undamaged cartilage tissue of the patient, proliferated in vitro, cultivated, and redifferentiated in the three-dimensional environment of a scaffold to support the extracellular matrix formation of the embedded chondrocytes. These constructs are used to replace the defect [1,2,3]. For this purpose, the scaffold material must meet high standards regarding biocompatibility, degradability, and the promotion of redifferentiation of the transferred cells. Scaffold materials for cartilage TE need to possess mechanical strength since cartilage is exposed to high loads. Since hyaline articular cartilage is not vascularized, nutrition of the cells is achieved by diffusion. To optimally enable the diffusion of nutrients and metabolic products, the constructs need to provide high permeability in addition to strength.

**Figure 1:**
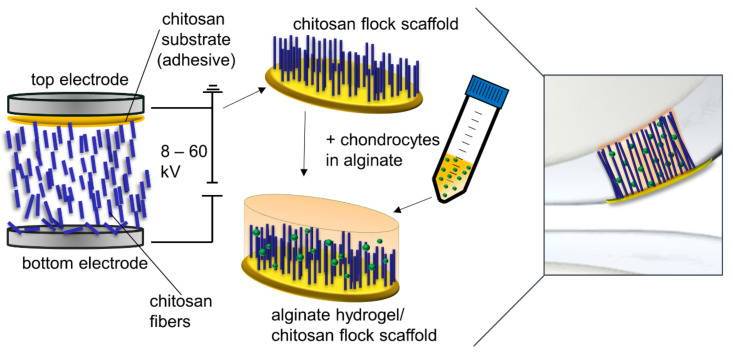
Principle of electrostatic flocking and scaffold production for chondrogenic tissue engineering. In an electrical field, 2 mm short wet spun chitosan fibers, at the beginning located on the bottom electrode, are shot vertically into a highly viscous chitosan layer covering the top electrode, which acts simultaneously as adhesive and substrate for the fibers. After drying, neutralization, and sterilization, the flock scaffold is seeded with chondrocytes in an alginate sol. The cells are immobilized after crosslinking in the combined alginate hydrogel/chitosan flock scaffold. After differentiation of the embedded cells, the scaffold is placed in a cartilage defect.

Various biopolymers such as collagen, alginate, hyaluronic acid, silk fibroin, and chitosan are investigated and applied for use in chondrogenic TE. The materials are processed to form membranes, hydrogels, sponges, and fibers, or additively manufactured structures [4,5,6]. In earlier studies, we developed a new scaffold type produced by electrostatic flocking of fibers, a process commonly used in the textile industry. Short fibers are applied vertically to a substrate which forms a membrane in the finished scaffold. In this process, short fibers are first accelerated in a high-voltage electrical field and applied to a substrate covered with an adhesive. The short fibers are uniformly distributed and vertically anchored in the adhesive layer in the finished scaffold (Figure 1). This process was already successfully implemented in biomedical applications several years ago with non-degradable polyamide fibers [7,8] as a multi-component system in combination with gelatin as adhesive and a collagen-based membrane as substrate. As a further development, chitosan was used for all components of the scaffold, and thus a fully resorbable flock scaffold was developed. This chitosan-based flock scaffold differs from other scaffolds for chondrogenic TE, commonly based on hydrogels or sponges, by showing a highly anisotropic morphology resulting in high porosity combined with good mechanical strength [9]. It was furthermore demonstrated that the anisotropic orientation of the fibers that mimic the orientation of collagen II fibers in the deep zone of articular cartilage leads to a favorable load-bearing capacity and elastic behavior [10]. 

Chitosan, which is frequently used for various TE purposes [11,12], provides the necessary biocompatibility and biodegradability. Due to the similarity of the polysaccharide structure to the glycosaminoglycans of cartilage, it is also well suited as a scaffold material for TE of cartilage, which is why it is often used for this purpose in addition to collagen and alginate [5,6]. Chitosan has been shown to promote the proliferation of stem cells and chondrocytes [13] and helps to maintain the chondrogenic phenotype in vivo and in vitro in a chitosan hydrogel [14]. Another study indicates an anti-apoptotic effect of chitosan degradation products on chondrocytes [15]. Alginate is a natural polysaccharide extracted from algae consisting of α-d-mannuronic acid and β-l-glucuronic acid residues. It was shown that alginate hydrogels support the redifferentiation of chondrocytes [16]. Hydrogels are an appealing biomaterial for TE because they form three-dimensional molecular networks with high water content, and this hydrophilic environment resembles the extracellular matrix (ECM) of tissues [5,17]. However, alginate hydrogels lack mechanical stability. 

Chondrocytes embedded in alginate beads release significantly less pro-inflammatory cytokines in the presence of chitosan [18], and composite chitosan–alginate scaffolds retained the spherical morphology and supported the expression of collagen II [19]. 

The optimal chondrogenic (re-)differentiation of harvested and monolayer-cultured chondrocytes plays an important role in any TE approach. Several studies have shown that chitosan used as a component of a scaffold for chondrogenic TE promotes differentiation, maturation, and spheroid formation of embedded cells [19,20,21]. Our first studies on chitosan-based flock scaffolds (CFS) were focused on mechanical properties and cytocompatibility. The aim of this study is to evaluate the suitability of CFS for cartilage TE for the first time. Flock scaffolds were combined with alginate hydrogels (ALG) and human primary chondrocytes. Chondrogenic (re-) differentiation and thus the suitability of flock scaffolds for cartilage TE was investigated in vitro.

## 2. Results

**Figure 2:**
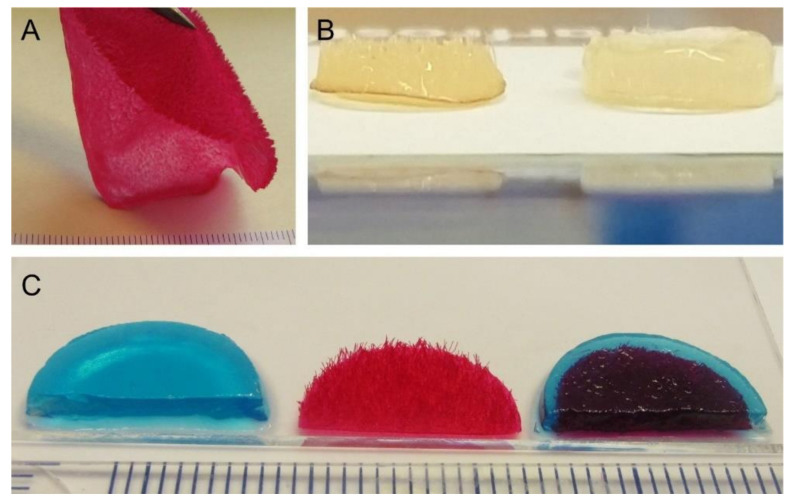
Images of different scaffold types: (**A**) Flocked chitosan source material after drying and neutralization in wet state, stained with Cibacron Brilliant Red 3B-A (CBr) for better visibility. (**B**) Chitosan flock scaffold without (left) and filled with alginate hydrogel (right) as used for cell culture experiments. (**C**) Pure alginate hydrogel scaffold (left), pure chitosan scaffold (middle), chitosan flock scaffold with alginate hydrogel (right). For a better representation after staining of chitosan with CBr and of alginate with Alcian blue. The scales on the rulers in (**A**,**C**) show mm, the scaffold diameter in (**B**) is 6 mm.

Three different types of scaffolds were included in the study: alginate hydrogels (ALG), chitosan flock scaffolds (CFS), and alginate-filled chitosan flock scaffolds (CFS+ALG; Figure 2). A detailed characterization of the CFS was published before [9,10]. The CFS used in this study showed a uniform distribution of the fibers with 2 mm length (fiber density 73 ± 8 mm^−2^) at a mean fiber distance of 149 ± 71 µm. For cell culture, 6 mm diameter scaffolds were punched out. After filling with 1.2% alginate solution in which the cells were suspended and crosslinking with calcium chloride solution, uniformly filled CFS+ALG were obtained (Figure 2b).

For the determination of the compressive strength, samples of 15 mm diameter were produced (Figure 2c). At a strain of 20%, no significant difference was found between the three groups. At 40 and 50% strain, the compressive strength of CFS and CFS+ALG was higher than that of the pure alginate gel, respectively. At 40% strain, it was 25.2 ± 3.5 kPa for CFS+ALG and 27.1 ± 0.9 kPa for CFS. The compressive strength for ALG was 8.4 ± 0.3 kPa at 40% strain.

**Figure 3:**
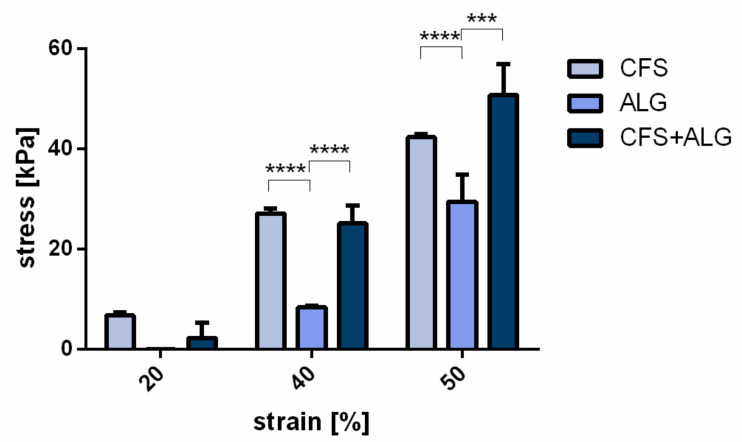
Mechanical characterization of scaffolds: Compressive strength of the chitosan flock scaffolds (CFS), alginate hydrogels (ALG), and chitosan/alginate flock scaffolds (CFS+ALG) were measured with a 100 N load sensor in wet state. Each sample had a diameter of 15 mm. The initial load was 0.1 N, and the compression rate was 5 mm/min. Compressive strengths were defined as the stress (kPa) at 20%, 40%, and 50% compression (strain) during the second of ten loading cycles to a maximum compression of 50%. Mean ± SD, n = 3. *p*-value **** < 0.0001, *** 0.0001–0.001.

At 50% strain, the compressive strength was highest in the CFS+ALG samples with 50.65 ± 6.3 kPa and significantly higher than the compressive strength in ALG (29.45 ± 5.3 kPa). CFS showed a compressive strength of 42.27 ± 0.65 kPa (Figure 3).

Primary human chondrocytes from three donors were introduced into the three types of scaffolds: CFS, ALG, and CFS+ALG. All cell-loaded constructs were cultured under chondrogenic stimulation for 21 days. After the first day, the uniform distribution of cells in the scaffolds was checked via confocal laser scanning microscopy (cLSM) after the medium change. 

**Figure 4:**
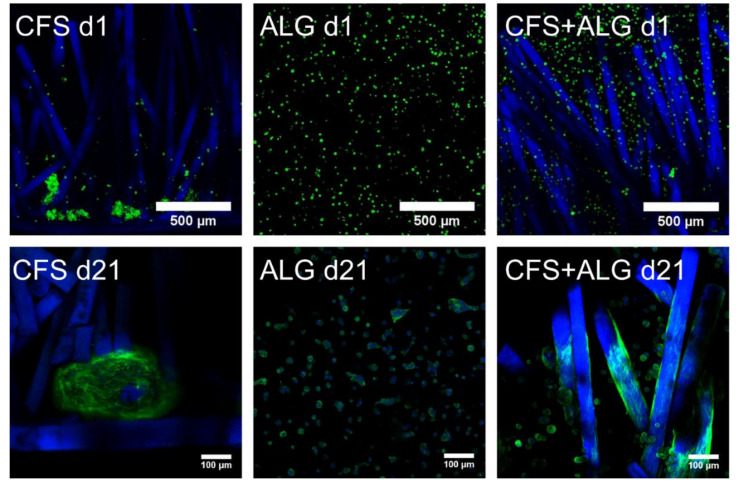
Representative cLSM images of chitosan flock scaffolds 1 and 21 days after seeding with human chondrocytes in chitosan flock scaffolds (CFS), alginate hydrogels (ALG), and chitosan/alginate flock scaffolds (CFS+ALG); Actin cytoskeletons of cells stained with Alexa-flour 488 Phalloidin (green), cell nuclei (stained with DAPI), and chitosan fibers appear blue because of autofluorescence. Reconstructions from cLSM image stacks. Scale bars represent 500 µm (day 1) or 100 µm (day 21), respectively.

Figure 4 shows the phenotype of chondrocytes introduced into the three scaffold types. On the first day, the cells were evenly distributed in the alginate gel and showed this even distribution also between the chitosan fibers. The alginate gel—and thus also the cells—completely filled the volume of the flock scaffolds. The cells were small, showed a round phenotype, and were separated in the alginate gel. In contrast to this, most of the cells in the CFS were located predominantly in the lower part of the scaffold, above and on the membrane. These cells already formed aggregates, single cells adhered along the fibers in the middle and upper part of the scaffold.

**Figure 5:**
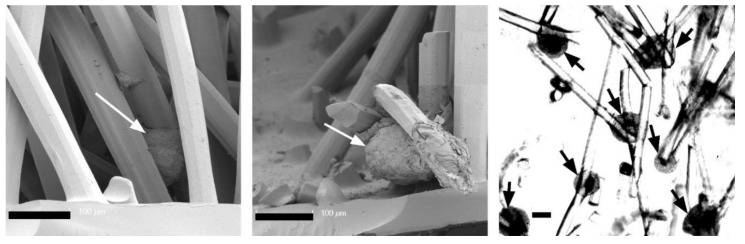
Formation of spheroids, 21 days after colonization with human chondrocytes in pure chitosan flock scaffolds (CFS) under chondrogenic cultivation conditions. Left/center: SEM micrographs of cross-sections, right: light-microscopic image. The formed cell aggregates are marked by arrows. Scale bars: 100 µm.

After cultivation for 21 days, the cells in pure alginate gel retained their round phenotype. After culturing the cells in CFS+ALG, some cells also exhibited a round phenotype in addition to cells with a spindle-shaped elongated cell shape that adhered directly to the chitosan fibers (Figure 4). In contrast, the cells in CFS formed spheroids about 100–200 µm in size, which were distributed over the entire scaffold and were mainly found near the basal chitosan membrane (Figure 5). 

**Figure 6:**
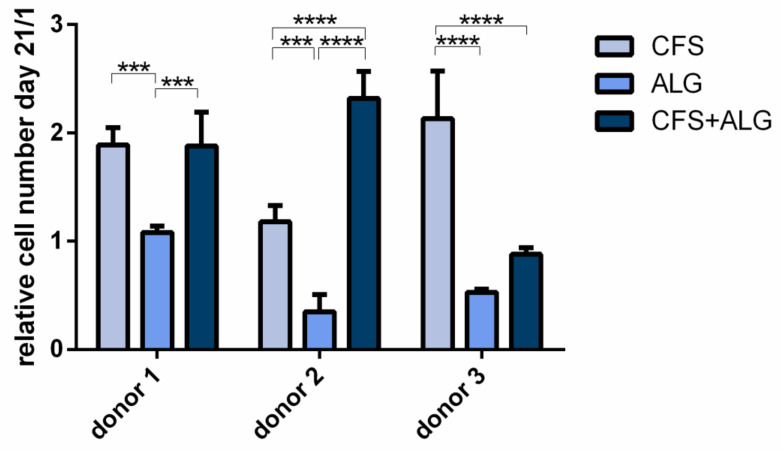
Proliferation of human Chondrocytes cultivated in chitosan flock scaffolds (CFS), alginate hydrogels (ALG), and chitosan/alginate flock scaffolds (CFS+ALG). Cells were seeded directly onto the CFS or within the alginate gel with a density of 4 × 10^6^/mL. Cell numbers were calculated from cytosolic LDH activities after 21 days of cultivation after cell lysis and are presented here relative to the cell number on day 1 after seeding. Mean ± SD, n = 3. *p*-value **** < 0.0001, *** 0.0001–0.001.

To evaluate the proliferation of chondrocytes in the different scaffold types, cytosolic lactate dehydrogenase (LDH) activity after cell lysis was determined after a cultivation period of 21 days and referred to the cell number determined on day 1 after seeding (Figure 6). For all three donors the proliferation was significantly higher in CFS than in ALG (*p*-value donor 1 and 2 < 0.001; donor 3 < 0.0001). The proliferation in CFS+ALG was significantly increased in the scaffolds seeded with chondrocytes of donor 1 (*p*-value < 0.001) and 2 (*p*-value < 0.0001) compared to ALG, while cells from donor 3 showed no significant difference between the two groups. When cultured in CFS, cells of all donors showed an increased cell number from day 1 to day 21 by 1.89 ± 0.16, 1.18 ± 0.15, and 2.13 ± 0.44-fold (donors 1, 2, and 3), respectively. In contrast, after cultivation in ALG, the number of cells from donor 1 remained equal at 1.08 ± 0.1-fold after 21 days or was reduced to 0.35 ± 0.16 and 0.53 ± 0.1-fold for donors 2 and 3, respectively. After cultivation in a CFS+ALG, the cell number increased to 1.88 ± 0.31 (donor 1) and 2.32 ± 0.25-fold (donor 2) while it decreased to 0.88 ± 0.1-fold for cells from the third donor. 

**Figure 7:**
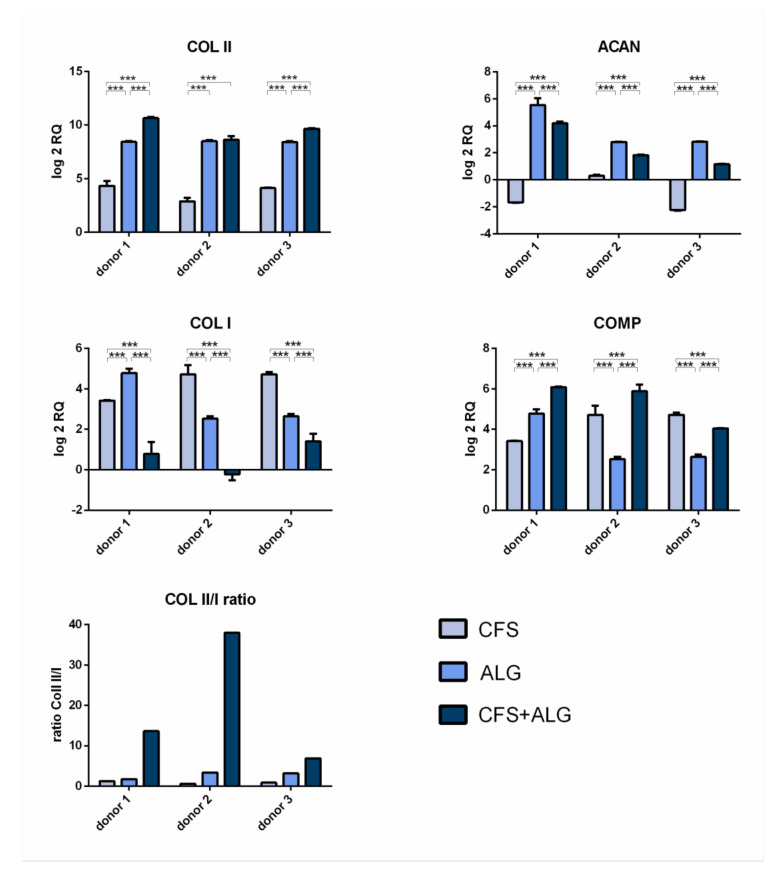
RT-qPCR analyses of mRNA of the chondrogenic genes Collagen II (*COL II*), Aggrecan (*ACAN*), and cartilage oligomeric matrix protein (*COMP*) and the dedifferentiation marker gene collagen I (*COL I*) after differentiation in chitosan flock scaffolds (CFS), alginate hydrogels (ALG), and chitosan/alginate flock scaffolds (CFS+ALG). Relative induction of gene expression was analyzed after 21 days of differentiation relative to *GAPDH* and human chondrocytes in monolayer culture at the end of expansion in passage 5. Ratios on mRNA levels were calculated from the relative quantification of collagen type II to I (*COL II*/I). Relative induction of gene expression was analyzed after 21 days of differentiation relative to *GAPDH* and human chondrocytes in monolayer culture at the end of expansion in passage 5. The results are shown as mean ± SD, n = 3. *p*-value *** 0.0001–0.001.

After 21 days of cultivation with chondrogenic stimulation, the expression of the chondrogenic marker genes aggrecan (*ACAN*), collagen II (*COL II*), and cartilage oligomeric matrix protein (*COMP*) was analyzed by quantitative real-time PCR (Figure 7). The expression of collagen I (*COL I*) as a fibroblast marker and, therefore, for chondrocyte dedifferentiation was also analyzed. Gene expression was determined relative to dedifferentiated cells after monolayer culture at the end of expansion in passage 5 and *GAPDH* as reference gene. 

The chondrogenic marker genes *COL II* and *COMP* show an increase in expression in all donors and in all scaffold types after 21 days of cultivation. The increased expression of *COL II* was highest in CFS+ALG (log2 RQ was between 8.6 and 10.6) and in ALG (log2 RQ 8.4–8.5) compared to chondrocytes cultivated in pure CFS (log2 RQ 2.8–4.3). The increased expression of *COMP* was shown in CFS+ALG at a log2 RQ value between 4.0 and 6.1, in ALG between 2.5 and 4.8, and in CFS between 3.4 and 4.7.

The expression of aggrecan varied depending on the scaffold type. While, after cultivation in CFS+ALG and ALG, all three donors showed an increase in expression (log2 RQ 1.1–4.2 and 2.8–5.5, respectively), the expression of *ACAN* was reduced (log2 RQ −1.7 and −2.3) or only slightly increased (log2 RQ of 0.3) after cultivation in CFS.

A low ratio of the expression of collagen II to collagen I provide an indication of possible dedifferentiation. While the expression of *COL I* in cells cultivated in ALG (log2 RQ 2.5–4.8) and CFS (log2 RQ 3.4–4.7) was increased, cultivation in combined CFS+ALG (log2 RQ −0.2 to 1.4-fold) showed mixed results. 

The collagen II/I ratio was highest for cells cultivated in CFS+ALG with 6.9, 13.6, and 37.9 for the different donors (Figure 7).

**Figure 8:**
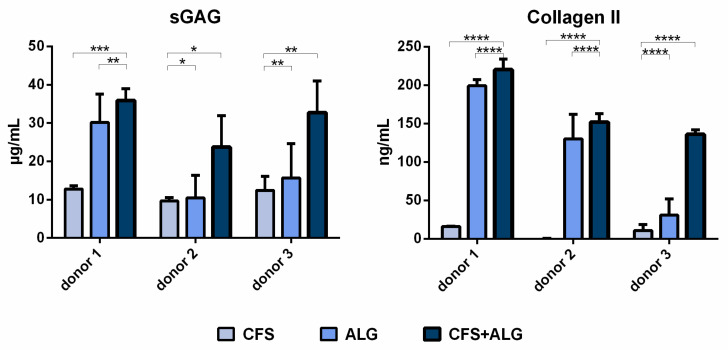
sGAG concentration in cell culture medium and collagen II in scaffolds, seeded with human chondrocytes of 3 donors 21 days after seeding in chitosan flock scaffolds (CFS), alginate hydrogels (ALG) and chitosan/alginate flock scaffolds (CFS+ALG). sGAG was determined using an assay based on Alcian blue, and collagen II was detected on protein level using ELISA. The results are shown as mean ± SD, n = 3. *p*-value **** < 0.0001, *** 0.0001–0.001, ** 0.001–0.01, * 0.01–0.05.

The secretion of sulfated glycosaminoglycans (sGAG) was higher for chondrocytes cultured in CFS+ALG compared to CFS or ALG alone (Figure 8). While the sGAG concentration in the cell culture medium of CFS+ALG scaffolds was 36 ± 3, 23 ± 8 and 32 ± 8 µg/mL for the different donors, a lower concentration of 30 ± 7, 10 ± 6 and 16 ± 9 µg/mL were detected in ALG, and the lowest concentrations of 13 ± 1, 10 ± 1 and 12 ± 4 µg/mL in CFS scaffolds. 

The secretion of collagen II was also highest (220 ± 14, 152 ± 11 and 136 ± 6 ng/mL, respectively) in CFS+ALG of all donors and significantly lower (16 ± 0.4 and 11 ± 7.5 ng/mL, respectively) or not detectable in all donors in CFS (Figure 8). The collagen II concentration after cultivation of cells from donors 1 and 2 in ALG was similar to that in combined scaffolds (199 ± 8 and 130 ± 32 ng/mL, respectively) and significantly lower with 31 ± 21 ng/mL for donor 3. sGAG and collagen II secretion relative to the respective cell numbers are displayed in Figure S1.

## 3. Discussion

Progress in tissue engineering and materials science offers a perspective to overcome current problems in the regeneration of damaged articular cartilage. One of these problems is the need for a scaffold material that provides sufficient mechanical strength, supports the redifferentiation of cultured chondrocytes, and shows high porosity for the exchange of nutrients and metabolites. The flock process, which is an established method in textile technology, can be adapted to produce scaffolds for tissue engineering, as demonstrated in our previous studies [7,8,9,10]. For the first time, we developed an anisotropic, single-material flock scaffold based on chitosan, which is suitable for tissue engineering due to its biocompatibility and biodegradability and demonstrated its applicability for the proliferation of hMSC and Saos-2 cells in vitro [9]. 

Since the properties of the scaffold (high mechanical strength in the direction of load, elasticity, and porosity) are promising for chondral TE, the aim of this study was to investigate the impact of these scaffolds on chondrogenic redifferentiation. For this purpose, human primary chondrocytes were cultivated in chitosan flock scaffolds in combination with an alginate hydrogel (CFS+ALG) under chondrogenic conditions and compared to the cultivation in alginate hydrogels (ALG) and chitosan flock scaffolds (CFS) alone.

Alginate is a polysaccharide composed of α-d-mannuronic acid and β-l-glucuronic acid derived from brown sea algae that is often used as a matrix for tissue engineering of cartilage [22,23] due to its high biocompatibility, low immunological stimuli, and ease of use, as it promotes the encapsulation of introduced chondrocytes and synthesis of cartilage-specific markers such as proteoglycans and collagen type II [6,16,23,24]. Hydrogels, as crosslinked networks containing 60–90% water, allow the transport of nutrients and the exchange of substances between the construct and the environment due to their inherent nanoporosity.

However, the low mechanical strength, which was confirmed in our results, impairs the application of pure alginate hydrogels for tissue engineering of cartilage, where high overall mechanical strength is required. Current approaches, therefore, aim to mimic the organization and structure of natural cartilage. The articular cartilage is anisotropic and inhomogeneous due to its microstructure. Its elastic properties are influenced by the arrangement of collagen fibers, which are oriented orthogonally to the bone–cartilage interface in the deep zone, randomly in the middle zone, and parallel to the surface in the superficial zone [2,4]. This anisotropy and associated resistance to loading is mimicked by the fiber alignment in the scaffold, which is also anisotropic as a result. Such an ordered structure can guide the introduced chondrocytes to produce their own typical extracellular matrix and thus fill a defect with functional tissue. Flocking creates an organized structure due to the vertical and parallel alignment of the fibers, resulting in an anisotropic scaffold with suitable mechanical stability and high elasticity. 

The low mechanical strength of the alginate hydrogel was significantly increased by combining it with a CFS, and the combination even exceeded the compressive strength of the pure flock scaffold. While in pure flock scaffolds, the high compressive strength is mainly due to the progressive elastic behavior of the fibers and the increased fiber-volume fraction as well as fiber-to-fiber contacts with increasing compression [10], combined scaffolds further benefit from the restricting gel matrix, which may decrease bending and buckling tendencies in individual embedded fibers and make the combined scaffold act more like a typical fiber-reinforced composite. 

Embedding of flock scaffolds into crosslinked alginate hydrogels results in fiber-reinforced hydrogels. Fiber reinforcement is a strategy to overcome the disadvantage of low stiffness of hydrogels while retaining their high water binding capacity and porosity at the same time [25,26,27,28]. Chitosan is a naturally occurring, positively charged polysaccharide that resembles the natural chemical structure of GAGs as a component of the cartilage matrix. The opposite electric charge of chitosan and alginate may also play a role in the increased compressive strength of the CFS+ALG, which is mediated by the negatively charged carboxylate groups of alginate and the positively charged amino groups of chitosan [29].

The deformation of up to 50% chosen in this study is significantly higher than the physiological deformations that occur, for example, in knee joint cartilage in vivo. Under load, the deformation of the knee cartilage is between 2 and 7% [30]. However, a higher deformation and multiple cycles were more suitable to characterize the material properties. Here, the values at 20, 40, and 50% strain provide a representative range of the scaffolds during the test. While the fibers still restructure during the first cycle, and water is released from the hydrogel due to the non-restraining test setup, this first cycle is not representative of the mechanical behavior of the scaffold. When implanting the scaffold into a defect, a lower Young’s modulus of a scaffold compared to the surrounding tissue increases its deformation; therefore, it is important to retain higher strains than those, which would occur in tissue in vivo. This behavior confirms the findings of Tonndorf et al., who have extensively studied the biomechanical properties of flock scaffolds [10].

It is known that cells embedded in alginate hydrogels maintain a round phenotype [5,16,24], which is important for the redifferentiation of the introduced chondrocytes, as they lose their round chondrogenic phenotype during two-dimensional in vitro expansion in monolayer culture [31]. In our study, the phenotype of the introduced cells differed in the three scaffold types. In the combined CFS+ALG, few cells adhered to the chitosan fibers with a fibroblast-like phenotype oriented along the fibers. Cell adhesion to chitosan was described for different cell types such as human bone mesenchymal stem cells (hBMSC), human adipose-tissue-derived stem cells, neurons, and fibroblasts [9,32,33,34,35] and depends on the degree of deacetylation of chitosan. Due to the positively charged amino groups and hydrophilic surface, chitosan enables interactions with the anionic cell surface and anionic GAGs, proteoglycans, and other negatively charged matrix molecules. 

In contrast to the alginate-containing scaffolds, the chondrocytes which were introduced into the pure CFS had aggregated into cellular agglomerates 24 h after seeding and formed large spheroids within 21 days. Cell aggregation and round morphology have previously been related to a pro-chondrogenic phenotype, and cellular aggregates enhance the chondrogenic differentiation ability of cells. It was previously shown that among other cell types, hBMSC and adipose-tissue-derived stem cells (hADSC) could self-organize into 3D spheroids with higher chondrogenic differentiation capacity when cultured on chitosan [36,37,38,39]. 

However, there are also studies in which hADSC and MSC retain a round phenotype during chondrogenic differentiation and adhere individually to chitosan forming an abundant matrix [33] or show a tendency to agglomerate [32]. Similar differences are reported in the cultivation of chondrocytes on chitosan. Rodrigues et al. [40] showed spread monolayer morphology of ATDC5 cells on chitosan membranes. Li and Zhang [19] compared chitosan and chitosan/alginate freeze-dried scaffolds; only the cells on the composite scaffold showed a round morphology, while they retained a fibroblast-like morphology on the pure chitosan scaffolds. In contrast, agglomeration of adherent chondrocytes with a round phenotype on chitosan/alginate fibers was demonstrated by Iwasaki [41]. Recent studies by Yeh et al. [38] and Rogina et al. [42] describe spontaneous spheroid formation on chitosan-based scaffolds for chondrocytes cultured under chondrogenic conditions. In the existing studies, parameters such as scaffold type (membranes, freeze-dried sponges, fibers, and thermogels), material topology, the nature of the neutralizing solutions, chitosan deacetylation degree (75–99%), molecular weight, combination with other materials (alginate, fibronectin, hyaluronic acid) and cell culture conditions differ. All these factors can influence the behavior of chondrocytes. In our study, spheroid formation may have been favored by cell aggregation and spatial proximity after seeding on the membrane because cell–cell contacts dominate over cell–matrix interactions in the formation of spheroids. In the CFS+ALG, cell–matrix contacts and cell–cell contacts are limited by immobilization. In vitro cultivation conditions of chondrocytes also influence the composition of the newly secreted extracellular matrix. In natural cartilage tissue, viscoelastic properties result from the structure and composition of this extracellular matrix, whose main components are proteoglycans and collagen II, both being arranged in a highly organized manner. Since 2D cultivation or expansion leads to dedifferentiation of chondrocytes [31,43], it is important that the cultivation conditions sustain redifferentiation with a chondrocyte-typical matrix. Alginate hydrogels have already been shown to support the growth and proliferation of encapsulated chondrocytes, as well as maintain their chondrogenic phenotype and lead to the expression of chondrogenic markers such as aggrecan, COMP, and sGAG, as well as collagen II [6,16,44]. Chondrocyte proliferation in the present study was lowest in the pure alginate scaffolds. The assay used quantitative measurement of the cytosolic enzyme lactate dehydrogenase (LDH). Since this assay analyzes the metabolic activity of the cells, its suitability to describe cell proliferation might be limited. In the majority of cases, LDH activity correlates with cell number [45]; however, changes in the differentiation state of the cells might also induce changes in LDH activity independent from the cell number. Further studies should involve real proliferation assays such as Ki67 staining. By determining RNA expression of chondrogenic differentiation markers as well as production of extracellular matrix compounds (sGAG and collagen II), our studies demonstrated successful chondrogenic redifferentiation of chondrocytes in the alginate hydrogel with and without chitosan. 

The highest expression of collagen II, cartilage oligomeric matrix protein, and sGAG was found after culturing chondrocytes in the CFS+ALG. At the same time, the expression of collagen I was lowest in these combined scaffolds. Collagen I is associated with a fibrocartilaginous and dedifferentiated cell type of chondrocytes and is observed to be increased after two-dimensional culturing for expansion or differentiation [16,31,43]. However, in line with our results, Caron et al. [16] described that collagen I is also induced upon three-dimensional cultivation of chondrocytes in alginate gels and cell pellets.

While collagen II expression at mRNA and protein levels of the chondrocytes cultivated in the CFS+ALG composite was similar to that in pure alginate, the low collagen I expression detected in CFS+ALG composites leads to a significantly increased collagen II/I ratio compared to both pure chitosan and alginate constructs. Marlovits et al. [46] reported that this ratio is above 400 at the mRNA level in freshly isolated chondrocytes and the beginning of cultivation and decreases to values between 0.1 and 1 during monolayer cultivation over 30 days. During cultivation in three-dimensional matrices, the expression of collagen II increases, whereas that of collagen I is decreasing; in our studies, the mRNA collagen II/I ratio showed a maximum of 37 after 21 days of redifferentiation of chondrocytes cultivated in CFS+ALG. 

Chondrocytes cultured in pure CFS showed the lowest expression of collagen II at the RNA and protein levels. Collagen II is important for chondrogenic differentiation, and it prevents hypertrophy of chondrocytes and supports the formation of cell–matrix contacts [47,48]. Although it is known that culturing chondrocytes in pellets or spheroids facilitates redifferentiation and is used to form hyaline-like cartilage [16,49] in our study, the differentiation of cells that formed spheroids in the CFS was low based on analyses on both RNA and protein levels. 

Several studies have focused on the expression of chondrogenic markers in scaffolds and hydrogels made of chitosan and alginate and have obtained heterogeneous results in detail. Li and Zhang [19] found a higher collagen II expression of chondrocytes in combined chitosan/alginate freeze-dried sponges compared to the expression of HTB94 in pure chitosan freeze-dried sponges. In vivo analyses of cell-laden alginate and chitosan hydrogels suggest higher suitability of chitosan for chondral tissue engineering since it retained the highest amount of sGAG and did not promote vascularization or endochondral ossification [14]. However, the results are only comparable to a limited extent due to the different types of scaffolds. Research of chondrogenic differentiation markers in combined chitosan/alginate scaffolds [29] showed that cartilaginous matrix proteins such as collagen type II, GAG, and aggrecan are produced when chondrocytes are cultured in these materials. Although these freeze-dried sponges, as well as pure alginate hydrogels, promoting the maintenance of the chondrogenic cell type, these scaffold types have low mechanical strength and degrade rapidly in physiological environments.

Especially in tissue engineering of cartilage, it is of particular importance to produce a graft that can withstand the multiple forces to which cartilage is subjected. The combination of alginate hydrogels with axially oriented chitosan fibers results in a mechanically stable anisotropic scaffold that can be more resistant to compressive loads perpendicular to the fiber orientation and has a higher elasticity compared to both the pure alginate hydrogels and flock scaffolds. Besides the promising properties of CFS in terms of biocompatibility, porosity, anisotropic morphology, and mechanical stability, these scaffolds were shown to support chondrogenic redifferentiation in our study. Differentiation of chondrocytes in an alginate hydrogel combined with a chitosan flock scaffold was superior to the pure alginate gel and pure chitosan flock scaffolds. By combining an alginate gel with its known advantages for chondrogenic differentiation with a chitosan flock scaffold, the disadvantages of the mechanical properties of a pure hydrogel can be overcome, and support of chondrogenic differentiation can be further improved. Further studies will be helpful to strengthen the evidence of this study, which is partially limited due to the small number of chondrocyte donors, the pathology of the donors, and the limited selection of chondrogenic markers. Moreover, further studies will involve in vivo testing of the constructs in an animal model.

## 4. Materials and Methods

### 4.1. Materials

Chitosan was purchased from Heppe Medical Chitosan, Halle, Germany, with a degree of deacetylation (DD) of 95% and a viscosity of 100 mPas (Chitosan 95/100) and 500 mPas (Chitosan 95/500), respectively, measured in a 1% chitosan solution in acetic acid as stated by the supplier. The molecular weight (*M*_W_) of chitosan 95/100 was between 100,000 and 250,000 g/mol, and for chitosan 95/500 was between 200,000 and 400,000 g/mol, as stated by the supplier. Alginate was purchased from Millipore Sigma, Darmstadt, Germany, as alginic acid sodium salt from brown algae (#71238). 

### 4.2. Scaffold Fabrication

The wet spinning of the chitosan filament yarn and the flocking of the fibers are described in detail elsewhere [9,10]. In brief, the spinning dope was prepared by mixing 8.5 wt% chitosan 95/100 and 2.81 vol% AcOH (acetic acid) in demineralized water and the spun yarn with a fiber diameter of 25 µm was cut into flock fibers with a length of 2 mm. The adhesive was prepared by mixing 5 wt% chitosan 95/500 and 5 vol% AcOH in demineralized water, stirring for 5 to 8 h, and aging for 24 h. 

For scaffold preparation, a thin layer of the chitosan adhesive (1 g made of 5 wt.% chitosan 95/500 and 5 vol.% AcOH) was evenly distributed on the top electrode (surface 26 cm²), and 0.5 g of cut fibers were distributed on the bottom electrode (surface 67 cm²) of an electrostatic flocking instrument (SPG 1000, Maag Flockmaschinen GmbH, Mössingen, Germany). 

By applying a voltage of 50 kV between the top and the bottom electrode, fibers were accelerated towards the top electrode and penetrated the adhesive (Figure 1). The resulting structure adhered to the top electrode and was immediately dried in an oven at 120 °C for 15 min and then detached from the electrode, neutralized in an aqueous 0.1 M NaOH solution with 10 vol.% ethanol, and subsequently immersed in 100% ethanol for 1 h and finally air-dried at room temperature.

Before use in cell culture, the dried scaffolds were soaked in distilled water for 30 min and round scaffolds with a diameter of 6 mm were punched out and steam sterilized while immersed in distilled water (121 °C, 20 min, D23 autoclave, Systec, Linden, Germany).

The scaffolds were transferred to a 70% ethanol solution and subsequently washed three times with phosphate-buffered saline (PBS; Gibco, Amarillo, TX, USA) and equilibrated in Dulbecco’s Modified Eagle’s Medium (DMEM; Gibco, Amarillo, TX, USA) with 10% fetal bovine serum (FCS; Corning, Corning, NY, USA) overnight. The medium was removed, and the scaffolds were washed with a cell culture medium and transferred to a 96-well plate for cell seeding.

To prepare the alginate solution, sodium alginate was autoclaved, and a 1.2 wt% solution was prepared using calcium-free DMEM high-glucose (4.5 g l^−1^ d-glucose; Gibco, Amarillo, TX, USA). Cells (see section cell culture) were resuspended in this alginate solution, and 50 µL was added either directly to a 96-well plate or to a chitosan flock scaffold (CFS) and then crosslinked with 100 mM calcium chloride solution for 30 min in an incubator at 37 °C. Excess calcium chloride solution was removed. The alginate hydrogels (ALG), the combined CFS+ALG, and the pure CFS were washed and incubated with a chondrogenic cell culture medium. 

### 4.3. Mechanical Analysis

Mechanical properties of the scaffolds were measured on a Z2.5 tensile tester (Zwick, Ulm, Germany) with a 100 N load sensor. The compressive strength of the scaffolds was measured in a wet state after soaking in Hanks’ Balanced Salt Solution (HBSS) with calcium and magnesium at pH 7.4 for 24 h. Each sample had a final diameter of 15 mm. For the combined CFS+ALG samples, round chitosan flock scaffolds with a diameter of 13 mm were punched out and embedded in alginate in a frame with a diameter of 15 mm. The initial load was 0.1 N, and the compression rate was 5 mm/min. Compressive strengths were defined as the stress at 20%, 40%, and 50% compression during the second of ten loading cycles to a maximum compression of 50%. 

### 4.4. Cell Culture

Human chondrocytes were obtained from the cartilage of the caput femoris, removed during endoprosthetic total hip arthroplasties at the University Hospital *Carl Gustav Carus* Dresden. Patients provided written informed consent, and the cell isolation was approved by the ethics commission of TU Dresden. 

Cartilage was cut into small pieces (1–2 mm) and incubated in 0.2% collagenase II in DMEM glutamax on a shaker at 37 °C for 15 h. The cell suspension was filtered through a 100 µm cell strainer, and cells were collected by centrifugation. The cell pellet was washed with PBS, centrifuged once more, and the cell pellet was resuspended in DMEM supplemented with 10% FCS, 100 U mL^−1^ penicillin, and 100 μg mL^−1^ streptomycin (Biochrom, Berlin, Germany), and the cells were afterward expanded in DMEM supplemented with 10% FCS, 100 U mL^−1^ penicillin, and 100 μg mL^−1^ streptomycin, cultivated in a humidified, 5% CO_2_ incubator at 37 °C.

Cells from 3 donors (age 53–61, 2 females, 1 male) in passage 5 were used for seeding of the scaffolds. For the CFS+ALG and the ALG controls, chondrocytes were resuspended in alginate sol at a concentration of 4 × 10^6^ cells/mL, and 50 µL of the alginate/cell suspension was added to the CFS or into a 96-well plate. For controls without hydrogel, 4 × 10^6^ cells/mL were resuspended in a cell culture medium, and 50 µL of cell suspension was added directly to the CFS. 

For chondrogenic differentiation, scaffolds were transferred to 24-well plates after 24 h and cultivated in chondrogenic differentiation medium consisting of DMEM high glucose (4.5 g L^−1^ d-glucose) supplemented with 100 U mL^−1^ penicillin, and 100 μg mL^−1^ streptomycin, 120 µM ascorbic acid 2-phosphate (AAP) (Sigma-Aldrich, St. Louis, MO, USA), 40 µg mL^−1^ l-proline (Sigma-Aldrich, St. Louis, MO, USA), 10^−7^ M dexamethasone (Sigma-Aldrich, St. Louis, MO, USA), ITS + 1 (Insulin-transferrin-sodium selenite + linoleic acid + bovine serum albumin; Sigma-Aldrich, St. Louis, MO, USA) and 10 ng mL^−1^ TGF-β3 (Miltenyi Biotec, Bergisch Gladbach, Germany).

### 4.5. Confocal Laser Scanning Microscopy (cLSM)

After rinsing the cells or cell-seeded samples in PBS with 2 mM calcium chloride (PBS+Ca) twice, the samples were fixed in 3.7% formaldehyde and permeabilized for 3 min using 0.1% Triton X-100 in PBS+Ca and then rinsed five times in PBS+Ca. Then, the autofluorescence of the samples was blocked by adding a 1% solution of bovine serum albumin (Sigma-Aldrich, St. Louis, MO, USA) in PBS+Ca. The nuclei of the cells were stained with 4′,6-diamidino-2-phenylindole (360 nM DAPI; Sigma-Aldrich, St. Louis, MO, USA) and the cytoskeleton with Alexa Fluor 488 Phalloidin (5 U/mL, Invitrogen, Waltham, MA, USA). The samples were rinsed three times in PBS+Ca and imaged using a Leica cLSM SP 5 (Leica, Wetzlar, Germany), provided by the core facility cellular imaging (CFCI) of the Medical Faculty of Technische Universität Dresden.

### 4.6. Scanning Electron Microscopy

After rinsing the cells or cell-seeded samples in HEPES with calcium and magnesium (HEPES Ca/Mg) twice, the samples were fixed in 2% glutaraldehyde in HEPES Ca/Mg followed by dehydration in graded series of ethanol and finally critical point drying (CPD 30, Bal-Tec, Balzers, Liechtenstein). All samples were fixed on carbon pads and sputter-coated with gold. A Philips XL 30/ESEM (Philips, Amsterdam, The Netherlands) with field emission gun operated in SEM mode was used for imaging.

### 4.7. LDH Activity

Cell proliferation was determined on day 1 and 21 after cell seeding through the activity of cytosolic lactate dehydrogenase (LDH), which reflects the number of viable cells. Frozen scaffolds were dissolved in an ice-cold ultrasonic bath using 55 mM sodium citrate solution with 0.9 wt% NaCl for 10 min and incubated on ice for an additional 30 min. Samples were then vortexed and centrifuged, and the supernatant was used to determine LDH or stored in low-binding tubes at −20° C for further analysis. 

LDH activity was quantified using The CytoTox 96 non-radioactive cytotoxicity assay (Promega, Madison, WI, USA) according to the manufacturer’s instructions. Absorbance was read at 492 nm in a microplate reader (Infinite 200 Pro, Tecan, Männedorf, Switzerland). LDH activity of the samples was correlated with the number of cells using a calibration line of defined cell numbers.

### 4.8. Quantitative Real-Time PCR 

After 21 days of chondrogenic differentiation, the alginate-containing samples were incubated with 55 mM sodium citrate solution with 0.9 wt% NaCl at 37 °C for 45 min and then thoroughly mixed. After centrifugation, the supernatant was discarded, and the RNA of the pellet was isolated using the peqGold MicroSpin total RNA Kit (VWR Peqlab, Erlangen, Germany) according to the manufacturer’s protocol. Samples without alginate (CFS and cell culture dish) were homogenized directly with the lysis buffer. RNA was quantified in a spectrophotometer (NanoDrop, Thermo Scientific, Waltham, MA, USA). Reverse transcriptase reactions were performed from 50 ng total RNA using Superscript II kit (Invitrogen, Waltham, MA, USA) with 200 U of superscript II reverse transcriptase.

Quantitative real-time PCR was carried out using 1.9 µL of c-DNA and TaqMan Fast Universal Master Mix (Applied Biosystems, Waltham, MA, USA) for the TaqMan Gene Expression Assays (ThermoFisher, Waltham, MA, USA) listed in Table 1. The reaction was performed on an Applied Biosystems 7500 cycler (Thermo Fisher, Waltham, MA, USA) with the following cycling profile: Polymerase activation at 95 °C for 20 s and 40 cycles of denaturation at 95 °C for 3 s and annealing at 60 °C for 30 s. For the calculation of the relative expression, the expression of the target genes was related to the housekeeping gene glyceraldehyde-3-phosphate dehydrogenase *(GAPDH)* and the expression of the chondrocytes after the end of the expansion in monolayer culture in passage 5 served as a control sample (day 0).

### 4.9. sGAG Quantification

Sulfated glycosaminoglycans (sGAG) were quantified in cell culture supernatants at day 21, taken 3 days after the last medium change, and stored at −20 °C. An assay based on the ionic interaction between Alcian blue and sGAG was used for the measurement according to the manufacturer’s protocol (Kamiya, USA #BP-004). For this, 50 µL of supernatant was incubated with 8 M guanidine-HCl and 50 µL of 0.54 M H_2_SO_4_ containing 7.5% (*v*/*v*) Triton-X 100. After the addition of Alcian blue, the solution was mixed and then centrifuged. The pellet was washed in dimethyl sulfoxide (DMSO) and then dissolved in 4 M guanidine-HCl containing 33% n-propanol and 0.25% Triton-X 100. Absorbance was read at 610 nm, and sGAG concentrations were calculated using a calibration line of chondroitin-6-sulfate from 12.5 to 400 µg/mL.

### 4.10. Collagen II ELISA

Collagen II was quantified as described elsewhere [50]. To determine the amount of collagen II in the scaffolds, 50 µL of the supernatant of the dissolved scaffolds (see Section 4.7) was used with a capture antibody mouse anti-chick collagen type II (1:2000, #7048; Chondrex, Woodinville, WA, USA) and a biotin-conjugated detection antibody mouse monoclonal anti-type II collagen (1:1000, # 7006, Chondrex) in an ELISA. Detection was performed using a streptavidin–horseradish peroxidase (# DY998; R&D Systems, Minneapolis, MN, USA) based conversion of the substrate 3,3′,5,5′-tetramethylbenzidine (#T4444; Sigma-Aldrich, St. Louis, MO, USA) and its photometric determination. The concentration was calculated using a calibration line of purified human collagen type II (# CC052; Millipore Sigma, Darmstadt, Germany).

### 4.11. Statistical Methods

Graphs show mean ± standard deviation. For the statistical evaluation, a two-way-ANOVA with a post-hoc Tukey test via Prism 6 software (GraphPad Software, San Diego, CA, USA) was performed. Analysis of relative gene expression data was based on a comparative CT method (ΔΔCT), and the relative expression was quantified and expressed as log2 RQ. The variance of the ΔCT is calculated from the standard deviations of three test samples/group and the control sample (day 0). A *p*-value < 0.05 was considered to be statistically significant. Statistical significance was evaluated using the following *p* values: *p* < 0.05 (*), *p* < 0.01 (**), *p* < 0.001 (***) or *p* < 0.0001 (****).

## 5. Conclusions

Chitosan flock scaffolds combined with alginate hydrogel synergistically enhance the differentiation of human chondrocytes. The application of biodegradable and biocompatible chitosan in the form of flocked fibers resulted in high compressive strength of the scaffold in the fiber direction, which was higher than that of the alginate hydrogel or flock scaffolds alone. This resulted in a scaffold with anisotropic morphology and mechanical properties, elasticity, and porosity that supported chondrogenic differentiation of inserted primary human chondrocytes and increased the expression of chondrogenic markers at the RNA and protein levels while maintaining lower collagen I synthesis than in a pure alginate hydrogel. As we showed in previous studies, by using the established textile engineering process of electrostatic flocking, the production of such scaffolds is simple, inexpensive, and the process parameters can be customized so that a scaffold with easily tunable properties can be manufactured. Based on the obtained data, the combination chitosan flock scaffold with alginate hydrogel is a promising new solution for articular cartilage tissue engineering and regeneration.

## Figures and Tables

**Table 1:** TaqMan Gene Expression Assays.

Gene Name	Gene Symbol	Assay ID
Glyceraldehyde-3-phosphate dehydrogenase	*GAPDH*	Hs02786624_g1
Collagen type I alpha 1	*COL1A1*	Hs00164004_m1
Aggrecan	*ACAN*	Hs00153936_m1
Cartilage oligomeric matrix protein	*COMP*	Hs00164359_m1
Collagen type II alpha 1	*COL2A1*	Hs00264051_m1

## Data Availability

All relevant data are included in the manuscript.

## Supplementary Materials

The following are available online at https://www.mdpi.com/article/10.3390/ijms22179341/s1.
