# Supplementary material for: Anisotropic Chitosan Scaffolds Generated by Electrostatic Flocking Combined with Alginate Hydrogel Support Chondrogenic Differentiation
Source: Int J Mol Sci. 2021 Aug 28;22(17):9341. doi: 10.3390/ijms22179341 (PMC8430627; doi:10.3390/ijms22179341)
Supplement: Supplementary file 1 [file ijms-22-09341-s001.zip › ijms-1327376-supplementary.pdf]

Supplemental material

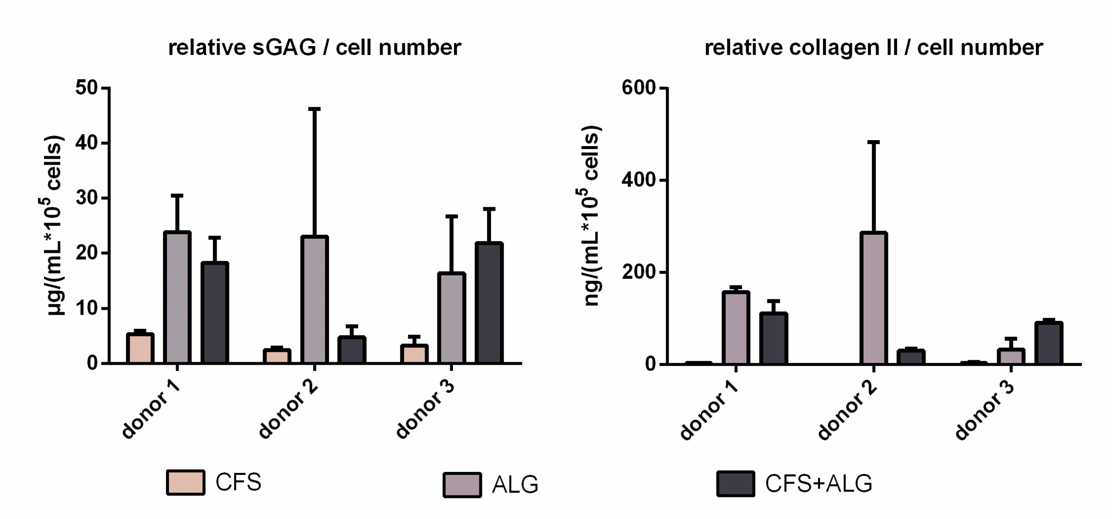

Figure S1. sGAG concentration in cell culture medium and collagen II in constructs relative to cell number on day 21, after seeding human chondrocytes in chitosan flock scaffolds (CFS), alginate hydrogels (ALG) and chitosan/alginate flock scaffolds (CFS+ALG). sGAG was determined using an assay based on Alcian blue, collagen II was detected on protein level using ELISA. The results are shown as mean ± SD, n= 3.
